# Short-Term Post-Harvest Stress that Affects Profiles of Volatile Organic Compounds and Gene Expression in Rocket Salad during Early Post-Harvest Senescence

**DOI:** 10.3390/plants9010004

**Published:** 2019-12-18

**Authors:** Natasha D. Spadafora, Giacomo Cocetta, Antonio Ferrante, Robert J. Herbert, Simone Dimitrova, Daniela Davoli, Marta Fernández, Valentine Patterson, Tinkara Vozel, Canesia Amarysti, Hilary J. Rogers, Carsten T. Müller

**Affiliations:** 1 School of Biosciences, Cardiff University, Sir Martin Evans Building, Museum Avenue, Cardiff CF10 3AX, UK; nspadafora@markes.com (N.D.S.); simon.dimitrova@nasekomo.life (S.D.); daniela.davoli91@gmail.com (D.D.); m.fernandez190@usp.ceu.es (M.F.); v.patterson02@gmail.com (V.P.); vozel.tinkara@gmail.com (T.V.); canesiadaa@gmail.com (C.A.); mullerct@cf.ac.uk (C.T.M.); 2 Markes International Ltd, Gwaun Elai Medi-Science Campus, Llantrisant RCT CF72 8XL, UK; 3 Department of Agricultural and Environmental Sciences, Università degli Studi di Milano, via Celoria 2, 20133 Milano, Italy; giacomo.cocetta@unimi.it (G.C.); antonio.ferrante@unimi.it (A.F.); 4 School of Science and the Environment, University of Worcester, Henwick Grove, Worcester WR2 6AJ, UK; r.herbert@worc.ac.uk

**Keywords:** *Diplotaxis tenuifolia*, NAC transcription factors, post-harvest senescence, rocket salad, vitamin C, volatile organic compounds

## Abstract

Once harvested, leaves undergo a process of senescence which shares some features with developmental senescence. These include changes in gene expression, metabolites, and loss of photosynthetic capacity. Of particular interest in fresh produce are changes in nutrient content and the aroma, which is dependent on the profile of volatile organic compounds (VOCs). Leafy salads are subjected to multiple stresses during and shortly after harvest, including mechanical damage, storage or transport under different temperature regimes, and low light. These are thought to impact on later shelf life performance by altering the progress of post-harvest senescence. Short term stresses in the first 24 h after harvest were simulated in wild rocket (*Diplotaxis tenuifolia*). These included dark (ambient temperature), dark and wounding (ambient temperature), and storage at 4 °C in darkness. The effects of stresses were monitored immediately afterwards and after one week of storage at 10 °C. Expression changes in two NAC transcription factors (orthologues of *ANAC059* and *ANAC019*), and a gene involved in isothiocyanate production (thiocyanate methyltransferase, *TMT*) were evident immediately after stress treatments with some expression changes persisting following storage. Vitamin C loss and microbial growth on leaves were also affected by stress treatments. VOC profiles were differentially affected by stress treatments and the storage period. Overall, short term post-harvest stresses affected multiple aspects of rocket leaf senescence during chilled storage even after a week. However, different stress combinations elicited different responses.

## 1. Introduction

*Eruca sativa* and *Diplotaxis tenuifolia,* both known as rocket salad, are becoming increasingly important commercially as part of fresh cut ready-to-eat salads and are appreciated for their sharp, spicy, and peppery flavor [1]. Moreover, nutritionally beneficial compounds including vitamin C, carotenoids, fibers, phenolic compounds, and glucosinolates are found in relatively high concentrations [2,3]. Leaves are frequently sold washed and ready to eat, sometimes in modified atmosphere packaging (MAP), and have a shelf life of up to 10 days at 5 to 10 °C [4,5].

The nutritional and sensory qualities of salad leaves need to be preserved during processing, packaging, labeling, storage, and transport after harvest [4]. Post-harvest stresses in rocket leaf production include cold during storage and transport, with variations in temperatures through the supply chain, dehydration, and mechanical damage during processing which activates wound responses in leaves [6]. Moreover, during much of the supply chain, salad leaves experience low light or dark conditions. The combination of these factors induces post-harvest senescence [7]. For example, photosynthetic efficiency fell significantly in rocket leaves after 24 h at 20 °C and 7 days at 4 °C [8]. Low temperatures (within an appropriate range) are important to maintain quality as they increase shelf life by lowering metabolic rates. This reduces degradation reactions that promote senescence [4,5,7,9]. Moreover, post-harvest storage affects metabolite content of rocket salad [10,11]. Glucosinolates are secondary metabolites which, in plants, have a protective role and are also responsible for the characteristic rocket peppery taste [12,13]. When plant tissues and cells are damaged by cutting or chewing, leaf glucosinolates are broken down to isothiocyanates by two enzymes, myrosinase and thiocyanate S-methyltransferase (TMT, [14,15]). Stress during harvest can alter the glucosinolate profile in leaves, [12,16] and depending on processing conditions, these compounds can lose their health-promoting properties and transform into anti-nutrients [17]. Glucosinolates are significantly reduced in rocket leaves after one or two weeks of storage at 4 °C [18]. However, transcriptomic analysis showed that *DtTMT* expression was upregulated by wounding, as well as cold and ambient dark stresses applied to leaves of *Diplotaxis tenuifolia* for 24 h following harvest [19]. This presumably increases the breakdown of glucosinolates into isothiocyanates on consumption. Processing and storage also affect microbial growth which could, in turn, affect flavor [6,9]. Specifically, increasing the storage temperature from the optimal 4 to 5 °C to 10 °C results in a rise in microbial growth on rocket leaves resulting in off odors [11,20]. 

The analysis of changes in volatile organic compounds (VOCs) can be a very sensitive tool to assess post-harvest changes in rocket salad [11,20,21,22]. VOC profiles were able to discriminate amongst fresh cut *Diplotaxis tenuifolia* leaves and those stored for two days or six days at 0 °C to 10 °C. Furthermore, rocket leaves stored under non-isothermal conditions in which a 5 °C storage regime was interrupted after two and six days with 24 h at 13 °C released higher levels of VOCs associated with off odors [22]. However, effects of different post-harvest stress combinations on rocket salad VOCs have not, to our knowledge, been investigated.

Plant responses to stress are dynamic and complex, involving changes at the physiological, biochemical, and molecular levels [23]. These responses are particularly complex when more natural, combined stresses are considered [24]. Stresses also increase the rate of leaf senescence and can initiate it prematurely [25]. Hence, stress can accelerate age-related deterioration of leaves, which influences shelf life and saleable quality of rocket leaves [7]. Thus, it is important to understand how leaves respond to post-harvest stresses, to determine which parameters are most important in restricting and predicting shelf life [5].

Stress responses are associated with changes in gene expression, and signal transduction pathways are triggered during stress responses, which vary depending on the time and the type of the stress [23,26]. Gene expression changes can be specific to each stress, however, there is crosstalk between stresses, which switches on a network of responses and involves tradeoffs between different stress responses [27]. NAC family transcription factors have a major role in responses to abiotic stress and senescence, because the expression of more than 20 of them increases during stress and senescence [28]. Nine NAC transcription factors were upregulated following exposure of *Diplotaxis tenuifolia* rocket leaves to a 24 h stress treatment immediately after harvest, including homologues of *Arabidopsis thaliana ANAC19* and *ANAC59* [19]. Both of these rocket *NAC* genes were upregulated in response to dehydration, wounding, dark, cold, and salinity. 

*ANAC19* has been associated with drought and salinity tolerance and with abscisic acid and methyl jasmonic acid signaling in *Arabidopsis thaliana* [28,29,30]. *ANAC19* can act individually in response to stress, but it is also implicated in overlapping stress responses [31]. *ANAC59* (also called *ORS1*) positively regulates leaf senescence in *Arabidopsis thaliana*, as overexpression accelerates senescence in transgenic plants. Furthermore, *ANAC59* expression is triggered by H_2_O_2_ and wounding [32]. In *D. tenuifolia*, *DtNAC59* was strongly upregulated in rocket leaves after just one day of post-harvest storage at 20 °C or seven days at 4 °C, and expression remained high for up to 14 days of storage at 4 °C [8].

Here, the effects of short-term post-harvest stresses on post-harvest senescence both immediately after harvest, and after a seven day storage period are reported. Stresses were selected to provide extreme examples of combined stresses experienced by rocket leaves during processing. Results show that some but not all effects of the stresses are still detected after the storage period and that different stresses result in different overlapping sets of responses. 

## 2. Results

### 2.1. Short Term Stresses Followed by Post-Harvest Storage Affected Physiological and Biochemical Parameters of the Rocket Leaves

**Figure 1:**
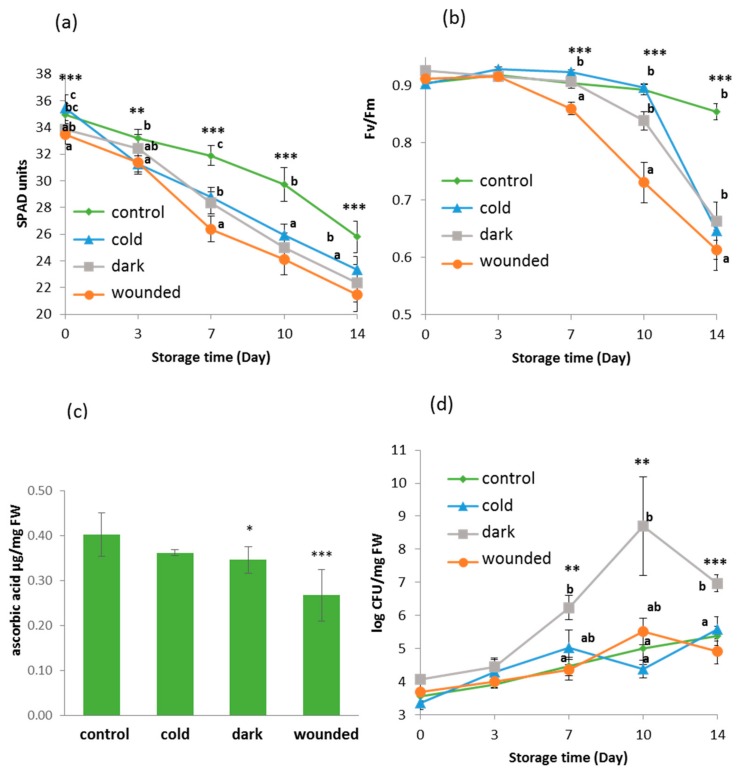
Physiological, biochemical characters, and microbial load in rocket leaves following 24 h stress treatments and storage at 10 °C in the dark. (**a**) SPAD meter reading, (*n* = 9, ± SD); (**b**) photosynthetic capacity (*n* = 12, ± SD); (**c**) ascorbic acid content (vitamin C) after 7 days of storage (*n* = 3, ± SD); and (**d**) microbial growth (*n* = 3, ± SD). Lower case letters indicate significant differences at each time point among treatments based on an ANOVA followed by a Tukey’s test; asterisks indicate differences to the control based on student’s t-tests (** *P* < 0.01 and *** < 0.001).

Chlorophyll fell gradually in all samples including the control over the storage period, although the control chlorophyll remained significantly higher than that of the stressed leaves from day seven of storage onwards (Figure 1a). Chlorophyll in wounded leaves was significantly lower than that of ambient dark or cold stressed leaves after seven days of chilled storage, and thereafter mean readings using a SPAD (soil-plant analyses development) portable chlorophyll meter remained lower but were not significantly different. Light use efficiency also fell in all samples after seven days of storage, and again in wounded leaves efficiency fell significantly more than in leaves subjected to the other short-term stresses (Figure 1b). In cold and ambient dark stressed leaves, Fv/Fm remained indistinguishable from the control up to and including day seven but on days 10 and 14 ambient dark values were significantly lower than the control or cold stored leaves. 

Vitamin C (ascorbic acid + dehydroascorbic acid) was significantly lower in ambient dark stressed and wound stressed leaves after seven days of storage at 10 °C. In contrast, vitamin C in cold stressed leaves was no different than the control (Figure 1c). 

### 2.2. Microbial Growth Increased in Dark Ambient Stressed Leaves Following Storage

In all samples, microbial growth rose with storage time, and was significantly higher in the ambient dark treated leaves after and including at seven days of storage. Other treatments did not have a significant effect on microbial growth (Figure 1d).

### 2.3. Gene Expression Changed During the Stress Treatment and Storage

**Figure 2:**
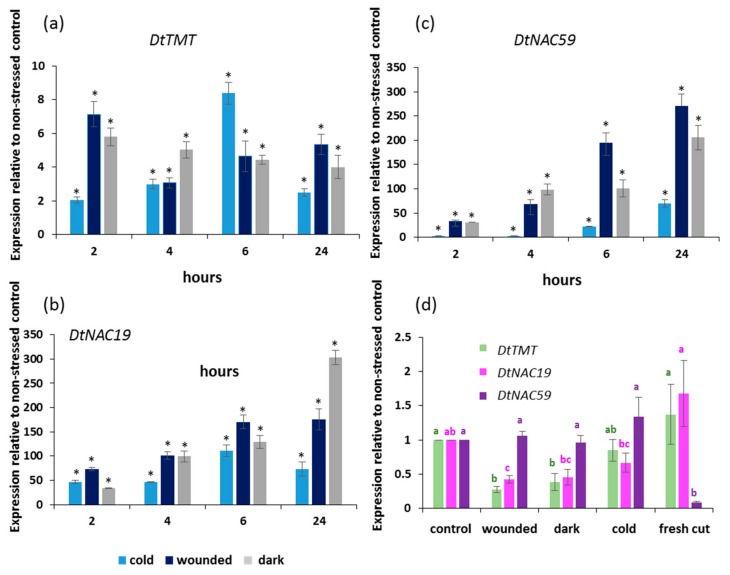
Gene expression in rocket leaves: (**a**–**c**) during 24 h stress treatments and (**d**) following 24 h stress treatments and storage at 10 °C in the dark for 7 days. (*n* = 3, ± SD). Lower case letters indicate significant differences at each time point among treatments based on an ANOVA followed by a Tukey’s test; asterisks indicate differences to the control at each time point based on Student’s t-tests (* *P* < 0.05).

Expression of *DtTMT* was analyzed throughout the 24 h stress period to assess whether stress affected its expression, and if so, whether the response was rapid or delayed. All three stresses upregulated *DtTMT* as compared with the unstressed control leaves, however, there were no clear differences in the speed of response amongst stresses (Figure 2a). Expression of two NAC transcription factors related to stress responses was also analyzed, and both *DtNAC19* and *DtNAC59* expression was upregulated in response to the stresses imposed (Figure 2b,c). However, the speed of response differed among stresses and between the two TFs. *DtNAC19* attained maximum expression 6 h after the start of the cold treatment, whereas *DtNAC59* reached maximum expression in response to cold after 24 h. Wounding response was maximal for both TFs after 24 h, as was the response to the ambient dark stress.

To assess whether these gene expression responses were transient, expression of the same genes was assessed following the 24 h stresses and seven days of storage at 10 °C. Here, a comparison of expression was made between the leaves that had not received the stress treatment but had been stored chilled for seven days, and the fresh cut leaves (Figure 2d). Compared to both fresh cut leaves and unstressed controls, both *DtTMT* and *DtNAC19* expression fell significantly in response to wounding and to ambient dark treatment. Post-harvest cold treatment did not affect *DtTMT* expression as compared with the controls. Cold treatment reduced expression of *DtNAC19* as compared with fresh cut leaves but did not reduce expression of *DtNAC19* as compared to leaves which were not stressed but were stored chilled. In contrast, *DtNAC59* expression was strongly upregulated by the post-harvest chilled storage with no significant effect of the imposed stresses.

### 2.4. VOC Profiles Were Affected by Short-Term Stress Treatments and Some Changes Persisted during Post-Harvest Storage

A total of 28 VOCs was detected across all the rocket leaf samples that were stress treated for 24 h (Table S1). These included isothiocyanates (seven), aldehydes (six), alcohols (four), nitrogen compounds (three), sulphur compounds, ketones and esters (two), one aromatic compound, and one alkyne. In contrast, after storage for seven days at 10 °C following the stress treatments, 30 VOCs were detected of which the most abundant were alcohols (seven) and aromatic compounds (seven) followed by esters (five), alkenes (four), terpenes (three), one isocyanate, and three VOCs which could not be identified at the family level based on a comparison to the NIST libraries (Table S2). Similar numbers of VOCs were detected following the four treatments (three stresses and control) both after 24 h and after the seven days of chilled storage.

**Figure 3:**
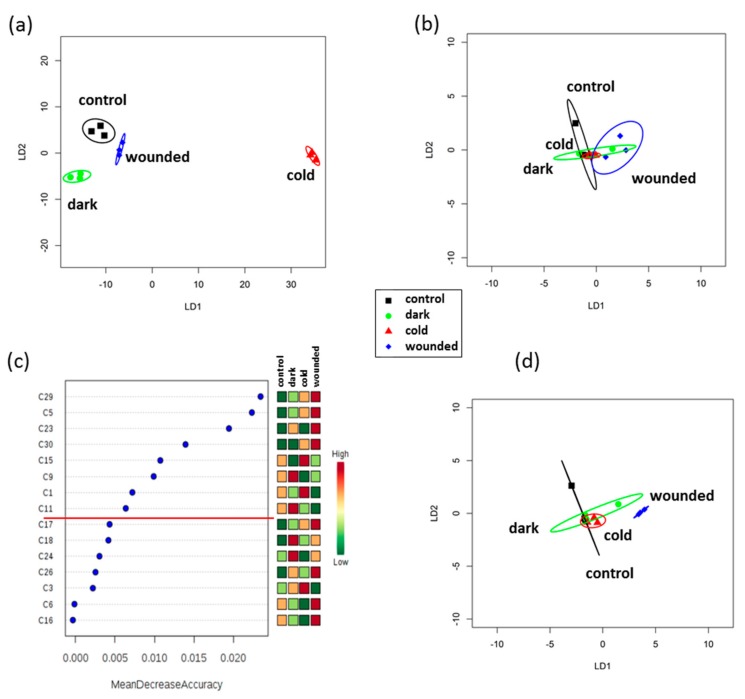
Analysis of VOC profiles from rocket salad using TD-GC-TOF-MS. A CAP model was produced for whole VOC profiles of leaves (**a**) subjected to three post-harvest stresses for 24 h, cold, wounding, and dark ambient (**b**) subjected to the same stress treatments but then stored for 7 days at 10 °C. (**c**) Random forest (as part of Metaboanalyst) was used to identify a subset of eight VOCs that were most discriminatory (above the red line) among the stress treatments in (**b**); the heatmap indicates the relative abundance of each VOC; (**d**) CAP model using only the eight most discriminatory VOC profiles from (**c**). The CAP plots use the first two linear discriminants (LD); each ellipse represents the 95% confidence interval. Percentage of correct classifications was (a) 83.3% (b) 41.7% (d) 50.0%, (*P* < 0.005, *n* = 3).

According to the relative abundance of the VOCs (Table S3), there was a significant effect of 24 h of stress treatment on the VOC profile (PerMANOVA, *P* < 0.01, R^2^ 0.488). Using canonical analysis of principal coordinates (CAP) to create linear discrimination plots showed good separation among the control and the different stress treatments with 83.3% correct classification (*P* < 0.05, Figure 3a).

Following storage at 10 °C for seven days, however, stress no longer had a significant effect on VOC profiles (PerMANOVA, *P* = 0.095, R^2^ 0.36, Table S4) and correct classification fell to 41.7% (*P* < 0.05, Figure 3b). To assess whether there were subtle changes in the VOC profile that were being masked by a largely unchanging profile, random forest analysis was applied. The following eight VOCs were identified as the most variable across the treatments (Table S2 and Figure 3c): 2-hexyl-1-decanol (C1), an unidentified branched alcohol (C5), 7-tetradecene (C9), a branched alkene (C11), 1,2,4-trimethylbenzene (C15), n-valeric acid ester (C23), and two unidentifiable compounds (C29 and C30). These eight VOCs showed a significant effect of stress treatment (PerMANOVA, *P* < 0.01, R^2^ 0.616). When the profiles from these eight VOCs were used in a CAP analysis, the correct classification was at 50% (*P* < 0.05). Moreover, they discriminated between leaves subjected to wound stress and all other stresses, as well as the control (Figure 3d).

## 3. Discussion

Evidence is presented for both short-term and long-term effects of stresses imposed immediately post-harvest in rocket leaves. Some of these effects are likely due to the accelerated initiation of senescence processes, while others may result from a specific stress response. The loss of chlorophyll pigmentation and the reduction in Fv/Fm during storage in darkness at 10 °C are expected, and previously reported [8,11]. However, there were also significant differences among the treatments. At the start of the storage period, SPAD readings indicated that wounded leaves were already less green than leaves subjected to other treatments, and the control. After seven days of storage, these differences became more evident. All stressed leaves received 24 h longer in darkness, which promoted chlorophyll loss [33]. However, the relatively greater loss in the wounded leaves could also be a result of the induction of ethylene production via 1-aminocyclopropane-1-carboxylic acid (ACC) biosynthesis that promotes senescence [34]. Wounding also induces ROS production, which has a role in accelerating senescence, although this has been shown to be light dependent [35]. Therefore, this is not likely to be involved here as the wounding was combined with dark storage for 24 h. Temperature seemed to have a less marked effect on chlorophyll loss as SPAD readings from cold/dark treated and ambient/dark treated leaves were not significantly different at any time point during storage. In ambient/dark treated leaves, there was a slightly greater reduction in Fv/Fm after 10 days of chilled storage as compared with the cold/dark treated leaves, although it was not statistically significant (*P* > 0.05). These results are in agreement with data on the effects of storage temperature on rocket salad [8,11]. The reduction in vitamin C found in wounded and dark/ambient treated leaves as compared with the control or the cold treated leaves also fits with a model in which temperature and wounding accelerate deterioration of the tissues and loss of vitamin C [36]. Similarly, in baby leaf spinach it has been reported that the ascorbic acid loss during storage, first, was affected by temperature and, then, intensified by cutting procedures [37]. Increased storage temperature was shown to accelerate vitamin C loss even after two days [11], although in other studies the reduction in ascorbic acid was slower [8]. This can depend on the physiological status and age of the leaves at harvest or the post-harvest treatment [36]. Microbial growth was greatest in the ambient/dark treated material showing a temperature effect, as previously reported [11]. Surprisingly, wounding did not increase microbial growth, despite evidence that wounding during processing promotes bacterial colonization [9], although the mechanical damage during commercial processing could be more complex. 

Physiological changes were reflected in VOC changes, as has been previously shown [11,20,21,22]. In agreement with previous studies [1,11], isothiocyanates were lost during storage. However, this finding contrasts with work in *Eruca sativa* where isothiocyanates rose during storage [38] and could relate to differences between the two rocket salad species. The differential changes in response to the different stress treatments is consistent with the subtle changes induced by storage at different temperatures [11] and effects of wounding on the release of VOCs [20,39]. These VOC changes indicate that wounding in particular has long-term effects on the bouquet, which could be important in considering post-harvest handling and salad processing. Moreover, the VOCs that were identified as having been affected after seven days of chilled storage could be potential markers for assessing the effects of different processing protocols. Previously, sulphur-containing VOCs associated with off odors were already detected within the first week of post-harvest storage at 10 °C but levels were much lower when leaves were stored at 5 °C [11,20]. These are thought to result from both tissue degradation as part of post-harvest senescence, and the action of spoilage microorganisms. Here, these VOCs were not detected even after the seven days of chilled storage, indicating that the leaves were in a very early stage of post-harvest senescence at this point.

Changes in gene expression were detected both during the 24 h stress period in response to the stress treatments, and also persisted after seven days of post-harvest storage. Transcriptomic data indicated that *DtTMT* was upregulated by all three stresses after 24 h [19,40]. Data here are in agreement but also indicate that the upregulation occurs much earlier, after only 2 to 6 h in the three stresses imposed as compared with the unstressed control. This is in agreement with the effect of salt stress on *DtTMT* expression in rocket leaves where again an upregulation of expression was noted in the first 2 h after the stress was imposed [40]. In *Arabidopsis thaliana*, the orthologue, *ATHOL1* (AT2G43910) is expressed throughout leaf development, but is downregulated in senescing leaves. This would fit with the reduced expression of *DtTMT* after seven days of storage, and although the control expression was not significantly reduced, expression in the ambient/dark and wounded leaves was reduced. This is in accordance with the greater loss of chlorophyll and photosynthetic capacity following these treatments indicating that they were entering senescence. 

As shown here through real-time PCR, transcriptomic data also showed an increase in the expression of *DtNAC19* and *DtNAC59* following 24 h of all three stress treatments [19]. Although both Arabidopsis *ANAC019* (AT1G52890) and *ANAC059* (AT3G29035) are upregulated during senescence, they are not upregulated in response to cold or wounding [41]. This suggests that the upregulation seen here was either due to early stages of senescence, or that regulation of the *DtNAC* genes differs from that of the Arabidopsis orthologues. Here, some interesting differences were also noted in the kinetics of the upregulation of *DtNAC59* during the 24 h of stress. The more rapid induction in response to wounding and ambient dark as compared with cold, could indicate an earlier activation of senescence under these treatments. Alternatively, if *DtNAC59* is also stress-activated, then the speed of response to wounding would be similar to early wound response genes in Arabidopsis where an upregulation of expression is detected already after 30 min [42]. The slower cold response is consistent with the later cold responses seen in many cold-responsive genes in Arabidopsis [43].

**Figure 4:**
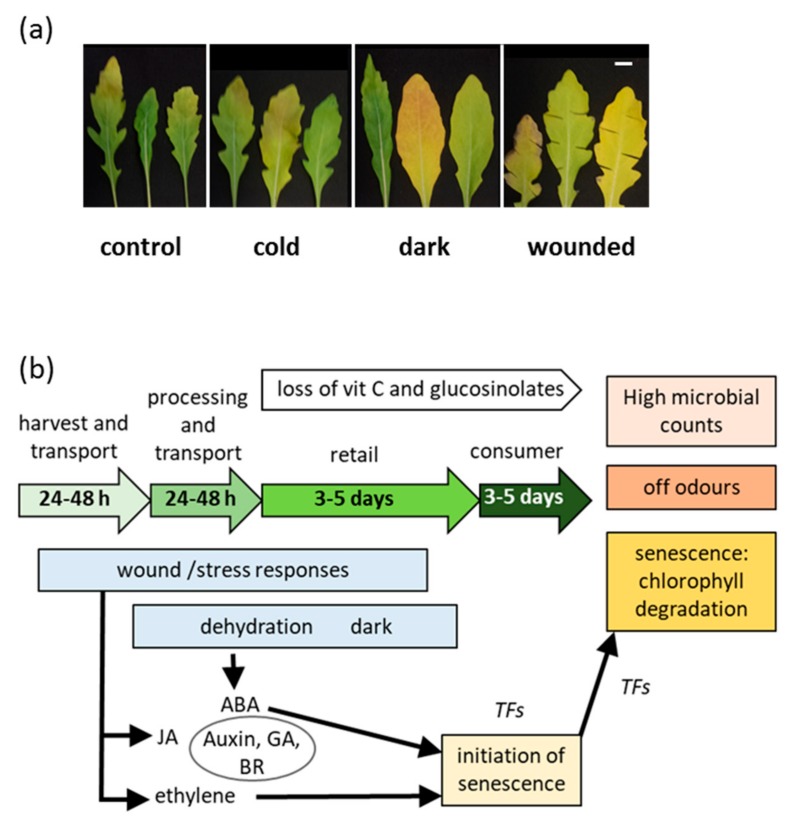
Interaction between stress and post-harvest senescence in rocket leaves (**a**) leaves after 24 h of stress treatments, followed by 7 days storage at 10 °C, (scale bar = 10 mm) and (**b**) model based on results presented and transcriptomic data from [19]. In the supply chain, processing imposes short term (typically 24 h) stresses involving wounding, temperature changes, and low light. Dehydration and dark/low light continue to affect leaves during transport, into retail, and in the consumer’s homes. The combined stresses activate responses including an initiation of senescence which results in chlorophyll breakdown and loss of key nutrients. The rise in microbial counts and tissue deterioration result in changes to the VOC profile which result in typical “off odors”.

The responses to early post-harvest wounding followed by chilled storage presented here, together with data from our previously published transcriptomic analysis of rocket under the same post-harvest stresses imposed here [19] can be used to construct a model, which considers the combined effects experienced by baby leaf salads in the supply chain (Figure 4b). A succession of stress responses to post-harvest handling result in changes to plant growth regulator homeostasis and the activation of stress responsive genes [19]. During chilled storage this is, then, followed by the initiation of senescence, which is detected by a reduction of photosynthetic capacity, vitamin C, and glucosinolates. At the same time, the increase in microbial populations combined with internal biochemical changes ultimately results in off odors and the termination of useful shelf life.

## 4. Materials and Methods 

### 4.1. Plant Material and Treatments

*Diplotaxis tenuifolia* L. var. Frastagliata plants were grown in compost for 30 days in a greenhouse at approximately 18 to 22 °C until several serrated leaves were present on each plant. Leaves were harvested and subjected to stress treatments according to [19]. Ambient and cold storage stress were imposed by storage for 24 h in darkness at 20 °C or 4 °C. Wounding stress was imposed by cutting each leaf from edge to midrib in 4 to 5 positions using scissors and stored for 24 h in the dark (Figure 4a). All leaves (15 to 20 g approximately) were stored in sealed plastic boxes. Controls consisted of leaves left on the plant for 24 h. Post-harvest storage was carried out for 7 to 10 days at 10 °C following the 24 h stress treatment. An additional control of fresh cut leaves, not subjected to storage, was used for the analysis of gene expression. All experiments comprised at least three biological replicates of at least three leaves. 

### 4.2. Microbiological Analyses and Assessment of Leaf Photosynthesis and Chlorophyll Content

Rocket leaf samples (0.1 g) were homogenized with 7.5 mL 1% (*w/v*) peptone water (Difco) with a mortar and pestle and the extracts filtered through Whatman filter paper. Serial dilutions were prepared with peptone water and 200 μL of extracts were plated in duplicate onto plate count agar (Sigma-Aldrich, UK) with appropriate serial dilutions to enable colony counting. All plates were incubated for 3 days at 25 °C. After incubation, colonies were counted and normalized to leaf fresh weight.

Chlorophyll content was assessed using a SPAD-502 Plus portable chlorophyll meter (Minolta, Osaka, Japan). Three measurements were taken on each leaf of each biological replicate. Chlorophyll *a* fluorescence-related parameters were measured using a MINI-PAM II (Pulse-Amplitude Modulation) portable chlorophyll fluorometer (Walz, Germany) where values were recorded for the parameters Fo (initial fluorescence), Fm (maximum fluorescence), and Fv/Fm (quantum maximum efficiency of photosystem II, PSII). Control leaves were dark acclimatized for at least an hour and readings were carried out in triplicate in the dark, avoiding veins. Care was taken not to wound or damage leaves during measurements.

### 4.3. Analysis of Vitamin C Content

Vitamin C extraction and analysis by HPLC was undertaken as described by [44], under low light and cold conditions. Metaphosphoric acid (5 mL of 5% *w*/*v*) was added to 1 g of ground rocket leaves and the mixture was homogenized by shaking and vortexing for 5 min, keeping on ice. Samples were centrifuged at 4700 g for 10 min at 4 °C, followed by filtration through Whatman 13 mm PVDF syringe filters, pore size 0.45 μm. 

HPLC was carried out using a SNA 4000 controller, an AS 300 autosampler, a P400 pump, SCM1000 degasser, and a UV 6000 LP photodiode array detector (Thermo Scientific, Waltham, MA, USA). Samples (20 μL) were analyzed isocratically over a 250 mm long 4.6 mm I.D. Phenomenex synergi polar (4 μm particle size, 80 Å pore size) column with polar “security guard” guard column, using 0.5% NaH_2_PO_4_ (pH 2.25 with H_3_PO_4_) acetonitrile (93:7 *v/v*) as mobile phase at a flow rate of 1.2 mL/min over 20 min. A calibration curve for ascorbic acid was obtained using standards of concentrations, 0.5 to 100 μg/mL. Each biological replicate was analyzed in triplicate. Data were analyzed using Xcalibur V 1.2. software (Thermo Scientific, Waltham, MA, USA). 

### 4.4. Collection and Detection of Volatile Organic Compounds

After the 24 h stress treatment and after seven days cold storage, rocket leaves were transferred to a sealed nalophene plastic bag (TJM Ltd, Barham, UK) and held at 20 °C for 90 min to equilibrate. Headspace (1000 mL) was collected using an Easy VOC hand pump (Markes International Ltd, Llantrisant, UK) onto SafeLok tubes (Markes international Ltd, Llantrisant, UK), that were packed with Tenax TA and SulfiCarb sorbents. VOC detection was undertaken essentially as described in [11]. Empty bag controls were included as blanks. VOCs were desorbed from tubes on a TD100 thermodesorption system (Markes International Ltd., Llantrisant, UK) for 5 min at 100 °C followed by 5 min at 280 °C, using a trap flow of 40 mL min^−1^. Trap desorption and transfer were carried out at 20 °C s^−1^ to 300 °C and a split flow of 5 mL min^−1^ into the GC (7890A, Agilent Technologies,). Samples were separated over 60 m, 0.32 mm I.D., 0.5 μm R × 5 ms (Restek) with 2 mL min^-1^ helium as a carrier gas under constant flow. The following temperature program was applied: initial temperature 40 °C for 2 min, followed by 5 °C min^−1^ to 240 °C, and a final hold of 5 min. Mass spectra were recorded from m/z 30 to 350 on a BenchTOF-dx, time-of-flight mass spectrometer (Markes International Ltd, Llantrisant, UK).

### 4.5. Analysis of Volatile Organic Compounds

VOC data were analyzed first using MSD ChemStation software (E.02.01.1177; Agilent Technologies, Cheadle, UK). They were deconvoluted and integrated using AMDIS (NIST11) and a custom retention-indexed mass spectral library. VOCs which were not detected in all three biological replicates of at least one sample were excluded. VOCs abundant in the blank controls were also excluded. Mass spectra were compared to the NIST 2011 library (using Stein, version 2.0 g, 2011, software). VOCs were only included in further analyses if they scored >80% in both forward and backward fit. Putative identification of VOCs was based on the mass spectrum and retention index (RI +/–15).

VOC data were analyzed essentially as described in [11]. Each VOC was normalized to the total peak area for each sample, and square root transformed. R software version 3.3.2 (R core development team 2015) was then used to perform permutational multivariate analysis of variance (PerMANOVA) and CAP analyses [45] using ”vegan” [46] and ”BiodiversityR” [47] packages. Ordination plots were generated, and 95% confidence intervals applied to the data points. Random forest analysis was used as part of Metaboanalyst [48].

### 4.6. RNA Extraction and Gene Expression Analysis

Leaves from each biological replicate were ground separately to a fine powder in liquid nitrogen and stored at −80 °C until use. Approximately 200 mg of leaf powder were used for each extraction and added to 2 mL of Tri reagent (Sigma, Darmstadt, Germany). Extractions followed manufacturer’s instructions, resuspending the final pellet in 100 μL of sterile water. RNA concentration was checked on a Nanodrop instrument and integrity was checked on a 1% agarose gel. Genomic DNA was removed using RQ1 DNase (Promega, Southhampton, UK) according to the manufacturer’s instructions, and removal was verified by PCR using DtEF1a primers (Table S1). cDNA was synthesized from 2 μg of RNA using oligo dT and M-MLV RNase H reverse transcriptase (Promega) according to the manufacturer’s instructions. RNA and cDNA were stored at −80 °C until used.

Real-time PCR was performed in a Roche Lightcycler using qPCRBIO Syber Green (PCR Biosystems, London, UK). Reactions were in 20 μL containing 30 ng of cDNA, 10μL of Sybergreen, 2.4 μL of sterile water, and 0.8 μL of each forward and reverse primer. Reactions were performed with three technical replicates on each of the three biological replicates with the following cycling program: 95 °C for 120 s, followed by 40 cycles of 95 °C for 30 s, 55 °C for 30 s, and 72 °C for 30 s followed by a melting curve from 55 to 98 °C to verify primer specificity. All primers used are listed in Table S5. Data were analyzed using the 2^−ΔΔct^ method [49].

## Supplementary Materials

The following are available online at https://www.mdpi.com/2223-7747/9/1/4/s1, Table S1: List of VOCs detected following 24 h stress treatments, Table S2: List of VOCs detected following 24 h stress treatments followed by 7 d of chilled storage, Table S3: Relative abundance of VOCs detected following 24 h stress treatments, Table S4: Relative abundance of VOCs detected following 24 h stress treatments followed by 7 d of chilled storage, Table S5: All primers used in this work for PCR.
